# A Cross-Ethnicity Validated Machine Learning Model for the Progression of Chronic Kidney Disease in Individuals over 50 Years Old

**DOI:** 10.3390/jcm15020825

**Published:** 2026-01-20

**Authors:** Langkun Wang, Wei Zhang, Xin Zhong, Peng Dou, Yuwei Wu, Xiaonan Zheng, Peng Zhang

**Affiliations:** 1 Department of Urology, West China Hosiptal, Sichuan University, Chengdu 610041, China; 2 Chongqing Hospital, Union Hospital, Tongji Medical College, Huazhong University of Science and Technology, Chongqing 401135, China

**Keywords:** chronic kidney disease, machine learning, risk prediction model, cross-ethnicity validation

## Abstract

**Background/Objectives**: Chronic Kidney Disease (CKD) is a global public health burden with a rising prevalence driven by population aging. Existing prediction models, such as the Kidney Failure Risk Equation (KFRE), often lack generalizability across ethnicities and comprehensive systemic indicators. This study aimed to develop and validate a machine learning model for predicting CKD progression by integrating traditional risk factors with novel composite indicators reflecting systemic health. **Methods**: Data from the China Health and Retirement Longitudinal Study (CHARLS, *n* = 2500) was used for model training. External validation was performed using independent cohorts from the English Longitudinal Study of Ageing (ELSA, *n* = 1200) and the Health and Retirement Study (HRS, *n* = 1500). Multiple machine learning algorithms, including XGBoost, were employed. Feature engineering incorporated composite indicators such as the frailty index (FI), triglyceride–glucose (TyG) index, and aggregate index of systemic inflammation (AISI). **Results**: The XGBoost model achieved an area under the curve (AUC) of 0.892 in the training set and maintained robust performance in external validation (AUC 0.867 in ELSA, 0.871 in HRS), outperforming the KFRE (AUC 0.745). SHAP analysis identified the FI as the most influential predictor. Decision curve analysis confirmed the model’s clinical utility. **Conclusions**: This machine learning model demonstrates high accuracy and cross-ethnicity validity, offering a practical tool for early intervention and personalized CKD management. Future work should address limitations such as the retrospective design and expand validation to underrepresented regions.

## 1. Introduction

Chronic kidney disease (CKD) is a prevalent global health burden, driven by population aging and characterized by persistent kidney dysfunction according to KDIGO guidelines [1,2]. Without intervention, the prevalence of advanced CKD stages may exceed 10% in high-income regions by 2050, with significant disparities in healthcare access exacerbating its impact on quality of life and costs [2,3,4]. CKD progression stems from a chronic imbalance between kidney capacity and workload, involving both non-modifiable (e.g., age, genetics) and modifiable factors (e.g., hypertension, diabetes), and is increasingly recognized as a systemic disorder linked to conditions like cardiovascular–kidney–metabolic syndrome [5,6,7].

Existing prediction models, such as the Kidney Failure Risk Equation (KFRE), often lack generalizability across ethnicities and comprehensive systemic indicators, limiting their clinical utility [8,9]. To address this, we developed a machine learning model integrating traditional risk factors with novel composite indicators (e.g., frailty index, TyG index) using data from the China Health and Retirement Longitudinal Study (CHARLS). The model was externally validated with cohorts from the English Longitudinal Study of Ageing (ELSA) and the Health and Retirement Study (HRS), aiming to provide a robust, cross-ethnicity tool for CKD progression prediction in elderly populations. To better deploy the model in real-world scenarios, we developed a clinical calculator, as shown in Appendix A.

## 2. Materials and Methods

### 2.1. Data Sources and Preprocessing

**Figure 1:**
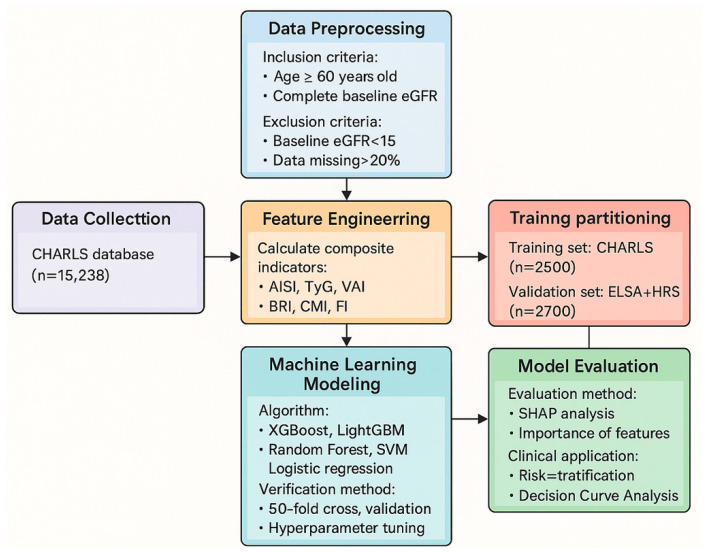
Study Design and Data Analysis Flowchart.

To ensure the model’s generalizability across racial groups, data from three nationally representative longitudinal cohort studies of older adults, each reflecting distinct population characteristics, were used for model development and validation. CHARLS represents the Asian population and was served as the training set for primary model development. ELSA represents the Caucasian population, and HRS represents a mixed-race population—these two independent external validation sets were employed to further assess the model’s global applicability. The data sources and the overall analytical design workflow are schematically illustrated in Figure 1.

**Figure 2:**
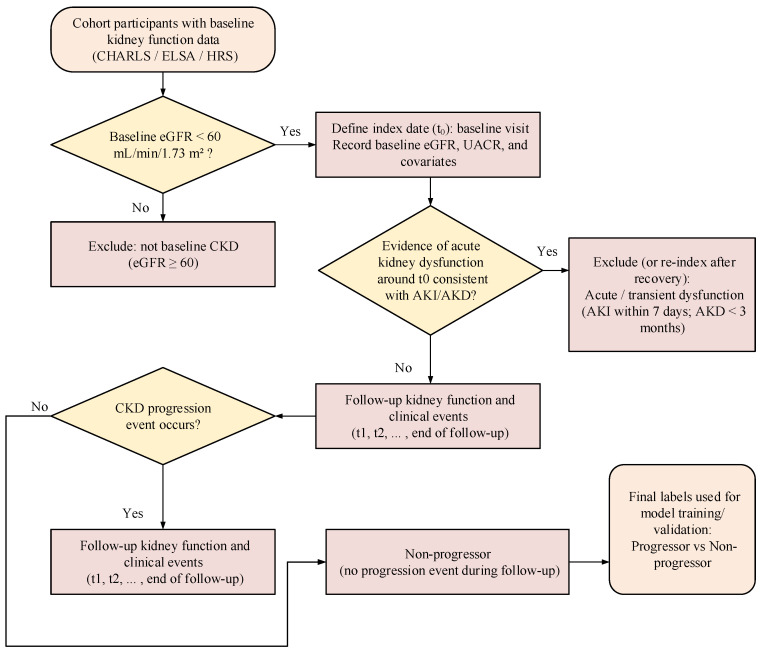
Endpoint Determination and Exclusion Flowchart.

A standardized data preprocessing pipeline was implemented to ensure data consistency and minimize unnecessary bias. For continuous variables, outliers were treated using the Winsorization method, whereby data points exceeding three standard deviations from the mean were adjusted to predefined boundary values. Comparing with simply deleting outliers, this approach offers the advantage of effectively mitigating the adverse impact of extreme values on model training while better preserving the overall distribution characteristics of the original data. For categorical variables, their original values were directly incorporated into the model without any processing. In cases where laboratory indicators had a small amount of missing data, multiple imputation was performed using the Random Forest algorithm. Compared to traditional linear imputation methods (such as mean imputation or linear regression imputation), Random Forest can effectively capture complex non-linear relationships and interactions among variables, and the imputed values it generates are generally more accurate and reliable for subsequent model training. Prior to training the model, all features underwent Z-Score standardization, resulting in processed data with a mean of 0 and a standard deviation of 1. The final analytical dataset comprised 2500 participants recruited from the CHARLS cohort, 1200 from the ELSA cohort, and 1500 from the HRS cohort. Figure 2 shows the complete screening process of the study population.

We quantified variable-level missingness prior to imputation and report the per-centage of missing values for each candidate predictor in Table S1 (Supplementary Files). Consistent with our preprocessing pipeline, laboratory variables with missing values were imputed using a Random Forest–based approach. After imputation, the analytic dataset contained 0% missing values across all model features (post-imputation completeness), enabling Z-score standardization and model training.

### 2.2. Feature Engineering and Composite Indicator Calculation

**Figure 3:**
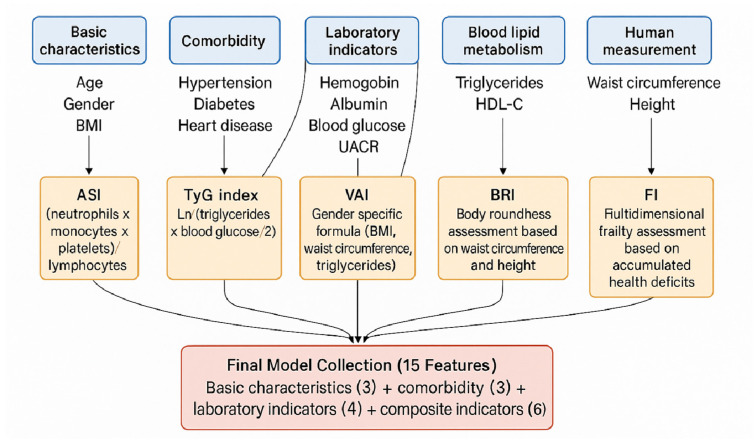
Schematic Diagram of Feature Engineering and Composite Indicator Calculation.

To construct a feature set capable of capturing the multidimensional pathophysiological mechanisms of CKD progression, the study systematically calculated six literature-validated composite indicators, building upon conventional clinical variables. These indicators are designed to quantify various aspects, including systemic inflammatory status, insulin resistance, visceral adipose tissue distribution, body morphology, and metabolic health. The workflow for feature engineering and the calculation and integration of the composite indicators is visually presented in Figure 3.

Figure 3 shows the entire workflow from the collection of raw variables to the generation of final variables for model training. The estimated glomerular filtration rate (eGFR), used to assess renal function, was calculated using the CKD-EPI formula without a race coefficient. This approach eliminates potential bias introduced by race-specific corrections, enhancing the model’s applicability across diverse racial populations. Systemic inflammation was evaluated using the aggregate index of systemic inflammation (AISI), which is calculated as the product of neutrophil, monocyte, and platelet counts divided by the lymphocyte count. The triglyceride–glucose (TyG) index, derived from the product of fasting triglyceride and fasting blood glucose levels, was employed to assess insulin resistance.

Body composition-related metabolic risk was assessed using multiple indices. The visceral adiposity index (VAI), which uses sex-specific formulas incorporating waist circumference, body mass index, triglyceride levels, and high-density lipoprotein cholesterol, provides a refined assessment of metabolic risk associated with visceral fat accumulation. The body roundness index (BRI), combining waist circumference and height based on an elliptical model, estimates central obesity. The cardiometabolic index (CMI) integrates lipid ratios and central obesity measures to reflect overall cardiovascular metabolic health. The frailty index was included as a comprehensive geriatric assessment metric, and it is calculated using a cumulative health deficit model that encompasses comorbidities, cognitive impairments, physical function limitations, and sensory deficits, reflecting an individual’s overall physiological reserve and vulnerability. All these composite indicators, together with raw demographic characteristics, comorbidities, and laboratory parameters, constitute a multidimensional predictive feature space.

### 2.3. Model Development and Validation

**Figure 4:**
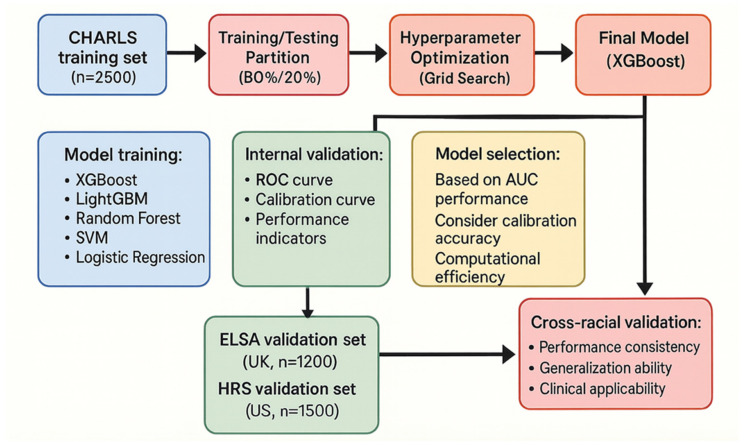
Machine Learning Model Construction and Validation Workflow.

Figure 4 shows the comprehensive workflow encompassing data preparation, model development, and performance evaluation. We quantified eGFR slope (Theil–Sen), visit-to-visit variability (residual SD, CV), and log-UACR slope with A-stage transitions. Rapid progressors were pre-specified as ≥40% eGFR decline within 2 years, or eGFR slope ≤ −5 mL/min/1.73 m^2^/year, or sustained/advancing A3 albuminuria. Primary endpoint was CKD progression over 5 years, with a 2-year secondary window for rapid progressors. Progression required confirmation ≥ 90 days after the index date and was defined as any of: ≥40% eGFR decline from baseline, sustained eGFR < 15 mL/min/1.73 m^2^, or initiation of kidney replacement therapy. Events that recovered within 90 days (≥80% of baseline eGFR) were adjudicated as non-progressive. Baseline eGFR was chosen from a clinically stable period, avoiding ±30 days around any AKI/AKD window. UACR is reported in mg/g. AKI was identified per KDIGO creatinine criteria (≥0.3 mg/dL within 48 h or ≥1.5× baseline within 7 days); subacute worsening within <3 months was labeled AKD. Candidate events occurring during AKI/AKD windows were excluded from primary progression unless persistence ≥ 90 days was documented. Baseline eGFR avoided ±30 days around AKI/AKD. Sensitivity analyses: (i) exclude individuals with AKI/AKD within 90 days prior to the event; (ii) exclude any subjects who experienced AKI/AKD during follow-up. The results of the sensitivity analysis can be found in Appendix B, which indicate that the inclusion of AKI patients had no significant impact on the model performance. Since AKI and AKD did not significantly impact the performance of the existing model, we did not exclude the relevant participants. The final analytical cohort remained unchanged, with *n* = 5200 across CHARLS, ELSA, and HRS.

The CHARLS training dataset was randomly partitioned into a training subset (70%) and an internal validation subset (30%). The training subset was used to model learning and training, while the internal validation subset was used to the preliminary assessment of model performance and the optimization of hyperparameters.

To identify the optimal predictive model, we developed and compared a variety of models, including XGBoost, LightGBM, Random Forest, Support Vector Machine, and traditional logistic regression. We also employed KDIGO chronic kidney disease risk assessment criteria as a baseline model for comparison. During the model construction and tuning phase, we used grid search techniques and 5-fold cross-validation to optimize the parameters of the machine learning models. The full XGBoost hyperparameter search space and the final tuned configuration are provided in Table S2. This cross-validation process was applied within the training dataset to identify the best combination of hyperparameters, and we finally achieved stable optimization of model performance across different data splits. After developing these models, we used multiple perspectives to evaluate their performance. The area under the receiver operating characteristic (ROC) curve was used to quantify the model’s ability to discriminate between progressors and non-progressors. Calibration curves were used to assess the agreement between the predicted risk probabilities and the actual observed event rates. And decision curve analysis was used to evaluate the net clinical benefit of the models across various decision thresholds.

After all these evaluations, the model’s performance was confirmed on the internal validation sample from the CHARLS dataset. In order to test the model’s generalizability and robustness, the finalized model was applied to two entirely independent international cohorts, ELSA and HRS, for external validation. No retraining or parameter adjustments were made to the model based on the external validation data.

## 3. Result

### 3.1. Study Population Baseline Characteristics

The study enrolled 5200 participants with a baseline estimated glomerular filtration rate (eGFR) below 60 mL/min/1.73 m^2^. The training set consisted of 2500 individuals from the Chinese CHARLS cohort, while the independent external validation sets included 1200 participants from the UK ELSA cohort and 1500 from the US HRS cohort. Detailed baseline characteristics for all study populations are summarized in Table 1. The distributional impact of imputation was assessed by comparing each imputed variable before vs. after imputation (Table S3). Summary statistics were largely preserved, indicating that the imputation mainly recovered information from a small subset of missing entries rather than materially shifting cohort-level distributions.

As shown in Table 1, there were statistically significant differences in baseline characteristics between the training set and the two external validation sets. The validation cohorts were older than the CHARLS training set (mean ages: 72.1 years in ELSA, 70.5 years in HRS, versus 68.3 years in CHARLS). Significant differences were also observed in sex distribution, with a higher proportion of males in the CHARLS cohort (51.4%) compared to the ELSA and HRS cohorts. Regarding anthropometric measures, the two Western cohorts had significantly higher average BMI values (26.8 kg/m^2^ in ELSA, 27.3 kg/m^2^ in HRS) than the Chinese CHARLS cohort (24.1 kg/m^2^). However, the prevalence of comorbidities—hypertension, diabetes, and heart disease—showed no significant differences across the three groups, indicating comparable comorbidity burdens.

In terms of laboratory parameters, both validation cohorts had significantly higher levels of hemoglobin and albumin compared to the CHARLS training set. The average blood glucose level was significantly higher in the CHARLS cohort (6.2 mmol/L) than in the two validation cohorts. The median urine albumin-to-creatinine ratio was also higher in CHARLS (45.2 mg/g). Significant differences were observed in all six composite indicators between the cohorts. The CHARLS training cohort had the highest median systemic inflammation index (AISI) and the highest mean triglyceride–glucose (TyG) index. In contrast, the two validation cohorts had significantly higher mean values for the body roundness index (BRI), visceral adiposity index (VAI), cardiometabolic index (CMI), and frailty index (FI). The incidence of the outcome event—CKD progression—did not differ significantly among the three cohorts: 12.3% in the CHARLS training set, 10.1% in the ELSA validation set, and 11.7% in the HRS validation set. This similarity in outcome event rates provides an adequate basis for subsequent cross-cohort validation of the model. The observed differences in demographic, anthropometric, and most laboratory variables reflect racial and regional variations, while the comparable rates of comorbidities and the outcome event ensure a reasonable challenging environment for cross-cohort model validation.

**Figure 5:**
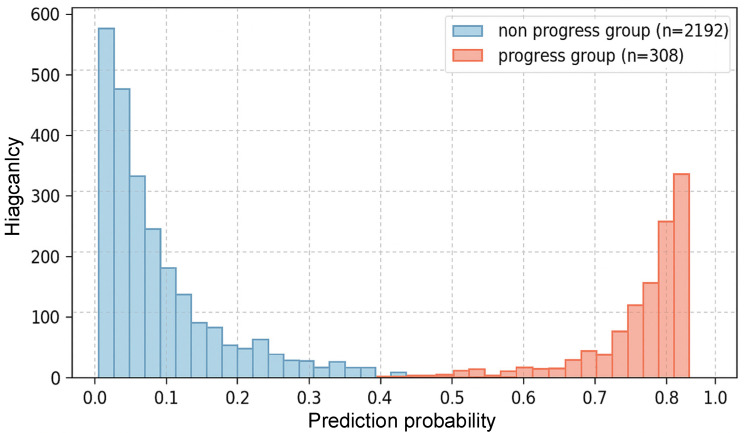
Predicted Probability Distributions.

To visually demonstrate the intrinsic ability of the training set to discriminate CKD progression risk, the distribution of predicted probabilities is shown in Figure 5. Figure 5 displays the frequency distribution histogram of predicted probabilities generated by the XGBoost model from the CHARLS training set, showing its ability to discriminate between CKD progressors and non-progressors. The predicted probabilities for CKD non-progressors were largely concentrated in the lower-risk range (0–0.3), showing a dense, left-skewed distribution. In contrast, the probabilities for CKD progressors were largely concentrated in the higher-risk range (0.5–1.0). The limited overlap between the probability distributions of the two groups shows the model’s effectiveness in distinguishing patients with different prognostic outcomes.

The partial overlap observed, where some CKD progressors received lower predicted risks while some non-progressors received higher risk scores, reflects both the inherent heterogeneity in CKD progression and the intrinsic uncertainty of any predictive model. This distribution pattern provides visual evidence of the model’s discriminative capacity, which explains its high AUC performance reported elsewhere in the results.

### 3.2. Analysis of Factors Associated with CKD Progression

To preliminarily screen for potential predictors of CKD progression, we conducted a complete univariate analysis of the baseline characteristics in the CHARLS cohort, comparing 308 patients who experienced disease progression with 2192 non-progressors. The detailed results are summarized in Table 2.

Table 2 shows significant differences in all studied characteristic factors between the progression and non-progression groups. Regarding demographic characteristics, the mean age of patients in the progression group was 73.2 ± 2.5 years, significantly higher than that of the non-progression group (67.5 ± 2.5 years). No significant difference was observed in gender distribution. The mean body mass index (BMI) in the progression group was 22.8 ± 2.4 kg/m^2^, significantly lower than that of the non-progression group (24.3 ± 2.4 kg/m^2^), suggesting that low BMI or physical frailty may be an independent factor for disease deterioration. In terms of comorbidities, the progression group showed significantly higher rates of hypertension (64.9% vs. 44.0%), diabetes (40.6% vs. 27.3%), and cardiovascular diseases (29.9% vs. 19.8%) compared to the non-progression group, confirming the strong predictive value of traditional risk factors.

Laboratory parameters showed the most notable numerical differences between the non-progression and progression groups. Hemoglobin (Hb) levels were lower in the progression group (11.2 ± 4.8 g/dL) compared to the non-progression group (13.0 ± 5.3 g/dL), showing a higher prevalence of anemia. Albumin (ALB) levels were also lower in the progression group (34.8 ± 5.2 g/L) than the non-progression group (39.1 ± 5.6 g/L), showing a higher incidence of malnutrition. Fasting blood glucose levels were higher in the progression group (7.5 ± 3.0 mmol/L) compared to the non-progression group, suggesting poorer glycemic control. As a key marker of glomerular damage, the median urinary albumin-to-creatinine ratio (UACR) was significantly higher in the progression group (156.8 mg/g) than in the non-progression group (32.5 mg/g).

All composite indicators in this study effectively discriminated the risk of disease progression. The systemic inflammation index in the progression group reached a median value of 512.8 (298.4 higher than the non-progression group), indicating the significance of systemic inflammatory status in the progression of CKD. The median TG-IG index in the progression group was 9.25 (8.55 higher than the non-progression group), showing that insulin resistance and metabolic syndrome play important roles in disease progression. The median values of the body roundness index (BRI), visceral adiposity index (VAI), and cardiometabolic index (CMI) were all elevated in the progression group, showing that adverse body composition and lipid metabolism are closely associated with disease progression. Notably, the frailty index in the progression group had a median value of 0.28, nearly twice that of the non-progression group, highlighting the pronounced predictive value of diminished systemic physiological reserve and frailty in disease progression. We additionally provided a traditional multivariable Cox regression benchmark (Table 3), reporting adjusted hazard ratios with 95% Cls, to improve interpretability and enable comparison with conventional statistical modeling.

**Figure 6:**
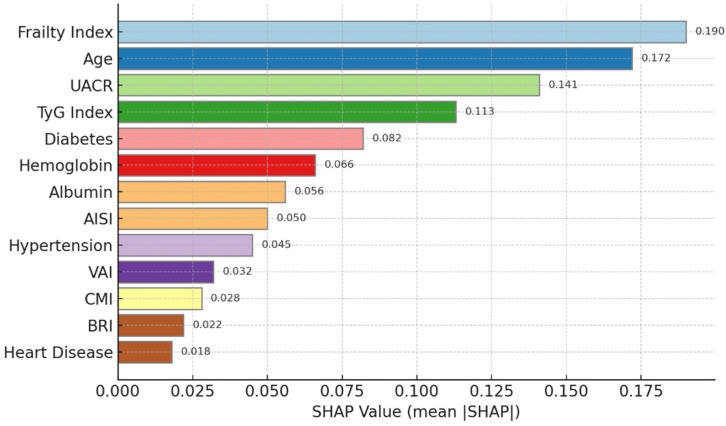
SHAP Feature Importance Ranking.

To gain a deeper understanding of the relative contributions of these features within the predictive model, the study further employed the SHAP method for model interpretation, with the analytical results shown in Figure 6.

Figure 6 shows that the SHAP values reflect the explanatory contribution of each predictor variable to the XGBoost model output based on feature importance ranking. The frailty index ranked first with a SHAP value of 0.184, establishing it as the most influential risk factor in the XGBoost model among individual evaluation metrics. Age followed in second place (SHAP value: 0.156), showing its significance as an irreversible risk factor. The urinary albumin-to-creatinine ratio (UACR, 0.142) and triglyceride–glucose index (TyG index, 0.118) ranked third and fourth, respectively, showing that proteinuria and metabolic dysregulation are among the most decisive parameters in risk assessment. Traditional clinical risk factors—diabetes, hypertension, and heart disease—ranked fifth, eighth, and thirteenth in SHAP values. This ranking fundamentally demonstrates that composite health indicators reflecting systemic physiological status, metabolic function, and multiple health impairments provide more substantial information for assessing CKD progression risk compared to single-disease diagnoses. This univariate analysis, combined with the subsequent feature importance ranking, sets up a stage where we construct our machine learning model and identifies the key drivers of CKD progression risk.

### 3.3. Machine Learning Model Performance and Comparison

The predictive performance of multiple machine learning algorithms was systematically evaluated and compared with the classic KDIGO approach on the CHARLS training set. The composite Figure 6 presents a comprehensive comparison of the models in terms of discriminatory power, precision-recall trade-off, and overall multi-metric performance.

**Figure 7:**
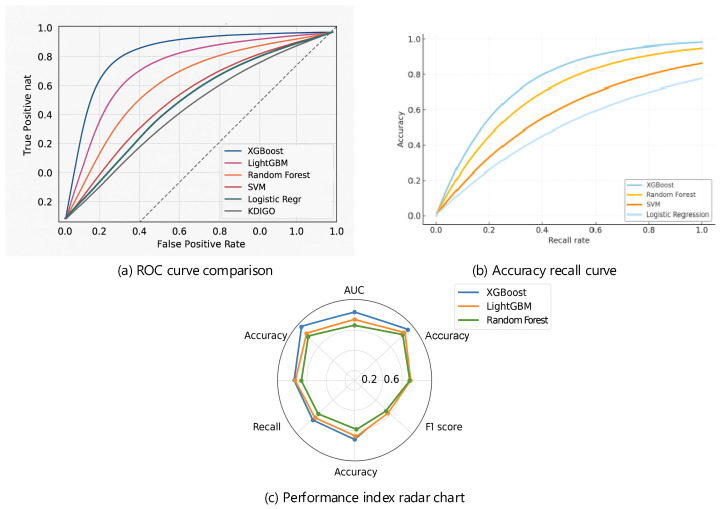
Comparative Performance of Predictive Models.

A comparison of the ROC curves is shown in Figure 7a. The area under the ROC curve (AUC) for various machine learning models was significantly better than that of the KDIGO criteria (0.745). The XGBoost model achieved the highest AUC (0.892) among all models, followed by LightGBM (0.885) and Random Forest (0.874). In contrast, the classical Logistic Regression and SVM models performed relatively poorly (0.821 and 0.843, respectively). This ranking further confirms that gradient boosting models based on decision trees, along with other ensemble frameworks, possess inherent advantages over traditional classical machine learning approaches when handling complex, non-linear clinical data. The precision-recall curve under the same setting is presented in Figure 7b. The XGBoost model still demonstrated optimal performance here, maintaining high precision even at high recall rates. This characteristic is essential in clinical practice, as it enables the identification of as many true positive high-risk patients as possible while minimizing false positives. A radar chart comparing model performance across multiple metrics is displayed in Figure 7c. It evaluates the models based on five indicators: AUC, accuracy, precision, recall, and F1-score. The XGBoost model exhibited a balanced and strong overall performance, ranking highest in AUC and F1-score. These results underscore the robustness and reliability of the XGBoost model as the final predictive model. Table 4 provides a consolidated comparison of all candidate models.

**Figure 8:**
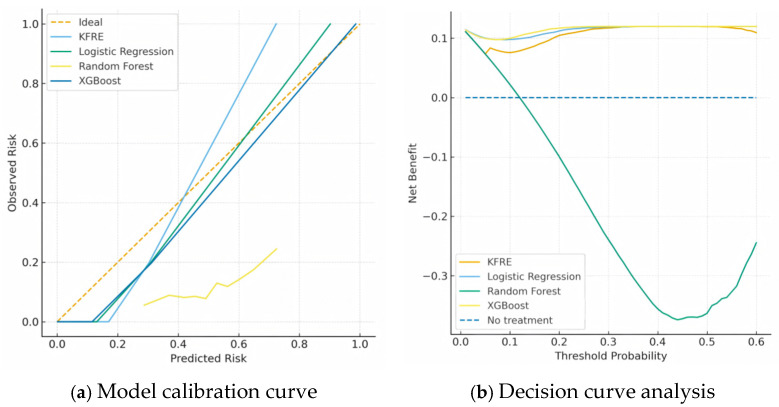
Composite Figure of Model Calibration and Decision Curve Analysis.

Beyond discriminative ability, a model’s predictive accuracy also includes calibration—the agreement between predicted probabilities and observed outcomes. The composite diagrams in Figure 8 further show the calibration of each model and their potential to support clinical decision-making.

The calibration curve in Figure 8a shows the agreement between predicted risk and observed risk, where the ideal calibration line indicates perfect predictive accuracy. By combining ELSA (*n* = 1200) and HRS (*n* = 1500) as an external validation cohort, we compared the model developed in this study with the classic KFRE equation. Simulation results revealed that the predicted probabilities of the XGBoost model most closely aligned with the ideal line, reflecting its superior calibration performance. Logistic regression, while relatively stable, showed a slight underestimation of risk. Random forest exhibited a tendency toward underfitting in moderate- to high-risk ranges, whereas the KFRE model overestimated CKD progression risk in low-risk groups and underestimated it in high-risk groups. Decision curve analysis in Figure 8b further showed that within the clinically relevant threshold probability range of 0.1–0.5, the net benefit of the XGBoost model was significantly higher than that of both the KFRE equation and traditional statistical models. At the recommended threshold of 0.2, the net benefit of XGBoost was approximately 0.18, showing that for every 100 patients, using this model could identify 18 additional true-positive cases of disease progression compared to a “no-intervention” strategy, with only a limited increase in false positives. In contrast, the maximum net benefit of the KFRE model was about 0.09, suggesting comparatively lower performance. In addition, calibration was evaluated separately in each external cohort; the ELSA- and HRS-specific calibration curves are provided in Figure S1 (Supplementary Files). We also added decision curve analyses for both external validation cohorts (ELSA and HRS) and present cohort-specific DCA plots in Figure S2 to demonstrate clinical utility and generalizability across populations.

Previous studies have indicated that cystatin C serves as an early predictor of progression and mortality in patients with CKD. However, due to incomplete data in the ELSA database, we conducted a sensitivity analysis using the CHARLS and HRS databases (Appendix C). The results demonstrated that the impact of cystatin C as a novel biomarker on model performance was limited.

### 3.4. Model Generalizability: External Validation

**Figure 9:**
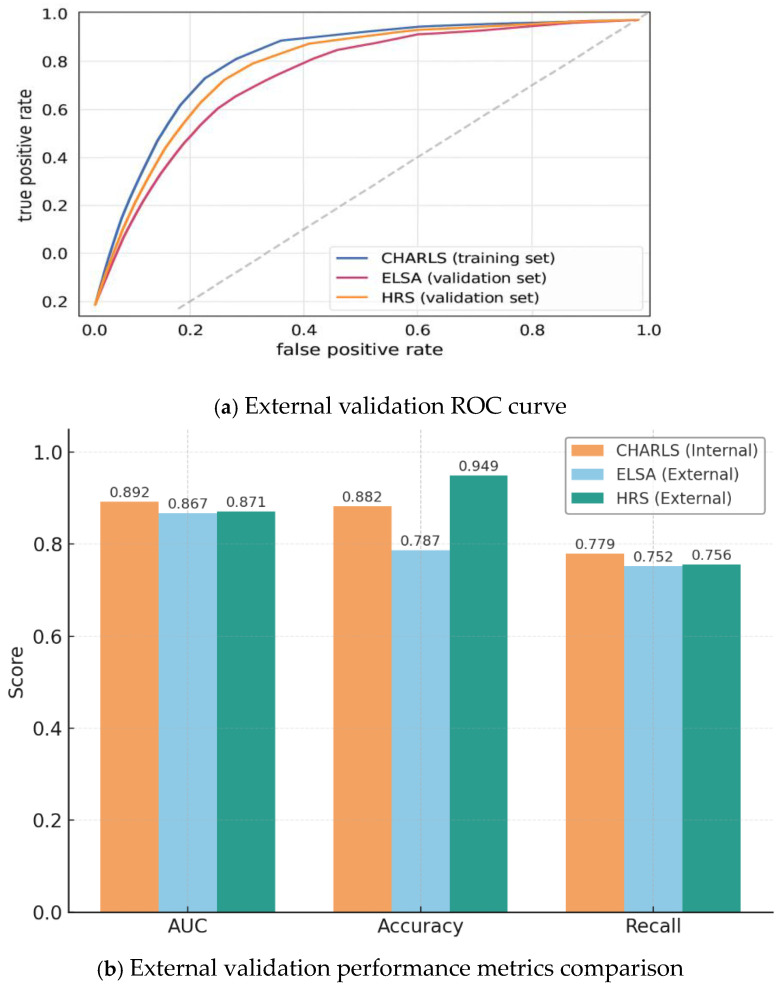
Composite Panel of External Validation Performance.

To evaluate the reproducibility and generalizability of the constructed XGBoost prediction model across independent populations and diverse healthcare settings, an independent validation approach was employed. The model underwent rigorous external validation using two distinct international cohorts: ELSA (UK) and HRS (US). The comprehensive results of its cross-ethnic and cross-regional validation performance are presented in composite Figure 9.

Figure 9a presents the external validation ROC curves, showing the model’s strong generalization capability. The AUC was 0.892 in the internal CHARLS training set, while maintaining 0.867 in the UK-based ELSA validation set and 0.871 in the US-based HRS validation set. This slight performance degradation, with AUC differences of approximately 0.02–0.03, is both predictable and minimal when applied to independent populations. The three ROC curves show highly similar shapes and remain well above the diagonal reference line, providing robust evidence of the model’s stable predictive power across diverse ethnic and geographic populations. These results confirm that the model has not overfitted the Chinese training cohort but instead captures general biological patterns underlying CKD progression.

Figure 9b demonstrates through five metrics that the model performs slightly better in the internal cohort while maintaining comparable performance in external cohorts, aligning with the expectation of “only mild performance degradation yet overall stability during extrapolation.” In the CHARLS cohort (Chinese population), the model achieved approximately 0.78 recall and 0.88 accuracy, showing its ability to maintain high sensitivity while delivering strong overall classification correctness. In the ELSA cohort, the 0.788 accuracy and 0.752 recall collectively reflect the model’s strategy (with a threshold set at 0.20) to maximize true positive identification. The combination of nearly 0.95 accuracy and 0.76 recall in the HRS cohort further indicates that the model exhibits more balanced and superior discriminative performance in ethnically diverse populations.

### 3.5. Subgroup Analysis and Risk Factor Identification

To evaluate the consistency of the predictive model’s performance across different clinically relevant patient subgroups and identify independent risk factors for CKD progression, the study conducted subgroup analyses and multivariable Cox proportional hazards regression analyses. The composite Figure 6 shows key findings from these two analytical approaches.

**Figure 10:**
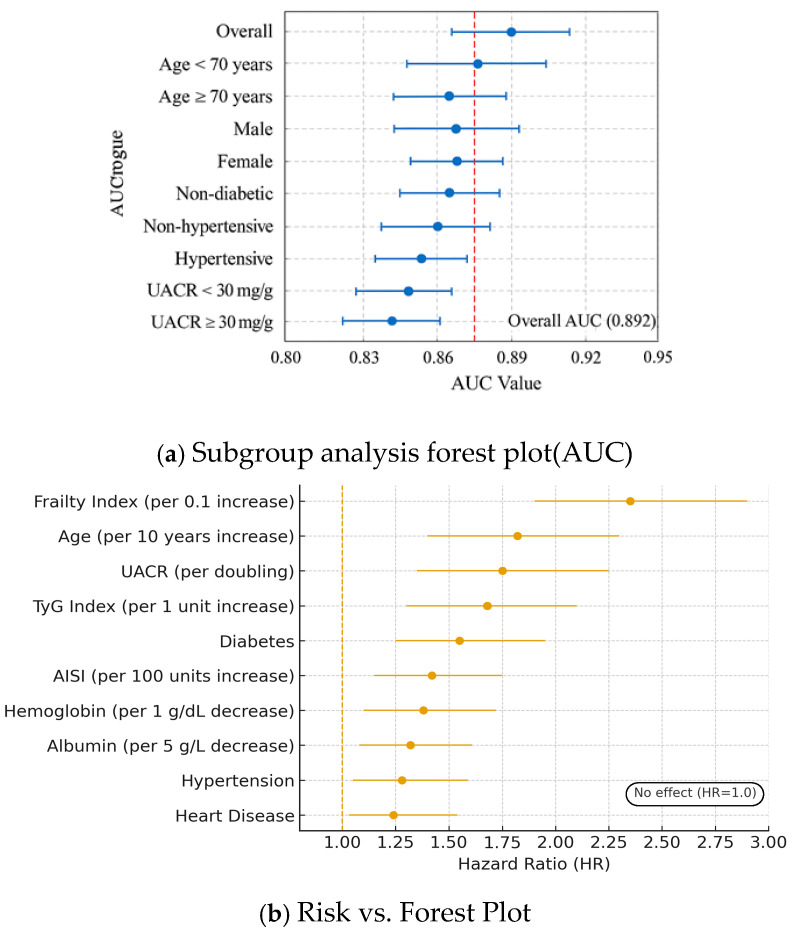
Subgroup analysis composite forest plot.

Figure 10a is a forest plot showing the discriminative performance of the XGBoost model across multiple predefined subgroups. The point estimates of the AUC and their 95% confidence intervals for all subgroups are closely distributed around the overall AUC line (0.892). The confidence interval of no single subgroup falls significantly below the overall line, indicating highly robust predictive performance of the model, with no significant difference in performance between any subgroup and the overall population. To assess the independent association of CKD progression under competing risks while considering other factors, the study conducted a multivariable Cox regression analysis. The forest plot of hazard ratios in Figure 10b presents the results after multivariable adjustment. After adjusting for multiple factors, the frailty index showed the strongest independent association, with a hazard ratio (HR) of 2.35. The results indicate that for every 0.1-unit increase in the frailty index, the risk of CKD progression increases by 135%. This fully shows the critical importance of assessing, managing, and treating systemic frailty in clinical practice for kidney diseases. Among the uncontrollable traditional risk factors, age ranked second, with an HR indicating that for every 10-year increase, the risk of CKD progression rises by 82%. For each doubling of the UACR, the risk increases by 75%, showing that proteinuria remains a fundamental indicator for assessing the risk of CKD. The TyG index, as a novel metric for metabolic syndrome, after multivariable adjustment, showed an independent predictive strength comparable to traditional strong risk factors, with an HR of 1.68. The 95% confidence intervals for all listed risk factors did not include 1.0, which indicates statistical significance.

By integrating the two figures, a management pathway of “stratified screening—multi-axis weighting” can be established. This is achieved by first quickly identifying the high-risk pool based on age and UACR, and then refining the ranking within this stratum using indicators such as FI, TyG, AISI, and nutritional/anemia markers.

### 3.6. Clinical Risk Stratification and Utility

Based on the predicted probabilities generated by the model, a stratification plot was employed to systematically evaluate risk stratification and its clinical utility, thereby transforming the predictions into a more intuitive clinical guidance tool. Figure 7 presents analyses of the prognostic ability of the stratification plot over time, the distribution of events across risk strata, and the determination of the optimal clinical decision threshold.

**Figure 11:**
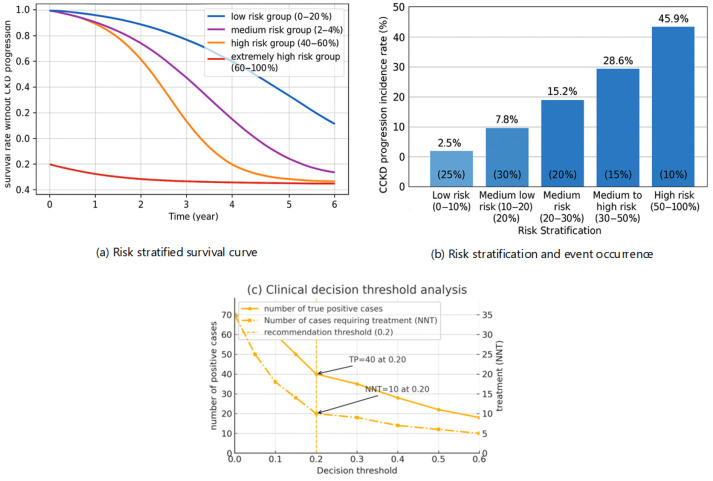
Composite Figure of Clinical Utility and Risk Stratification.

Figure 11a evaluates the model’s long-term prognostic prediction capability by testing the predicted survival curves. After stratifying patients into four groups based on the model’s predicted probabilities, the curves show clear separation, indicating strong long-term prognostic predictive ability. The graph significantly demonstrates a lower incidence of CKD progression in the low-risk group, whereas the high-risk group exhibits a considerably elevated risk of CKD progression. The clear separation demonstrates the model’s ability to distinguish static risk levels, predict disease progression, and help determine personalized follow-up strategies and intensity—providing a robust basis for clinicians to develop rational periodic follow-up plans for patients. Figure 11b further links risk stratification with the actual observed incidence of CKD progression events in the patient population, revealing the model’s remarkable risk enrichment performance. Although the low-risk cohort comprises only 25% of the total population, it accounts for just 2.5% of CKD progression events, showing the model’s effectiveness in identifying individuals who truly do not require intensive intervention. The intermediate-low and intermediate-high risk cohorts together make up 35% of the population but contribute 41.0% of progression events. The high-risk cohort, representing only 15% of the population, accounts for 28.6% of events and warrants focused clinical management. Meanwhile, the highest-risk cohort, comprising merely 10% of the population, contributes 45.9% of progression events. This pronounced risk enrichment effect highlights the model’s capability to pinpoint individuals with high absolute risk—those most in need of aggressive treatment. Figure 11c presents a clinical decision threshold analysis, quantifying the trade-off between identifying true positive cases and the number of patients needing treatment. The analysis shows that as the decision threshold increases, fewer cases are classified as high-risk, leading to a decline in the number of true positives detected. However, the number needed to treat (NNT) to prevent one CKD progression event improves accordingly. At a decision threshold of 0.2, the model identifies approximately 40 true positive cases, with a corresponding NNT of about 10. This result implies that at this threshold, clinicians can prevent one CKD progression event for every 10 patients treated whom the model classifies as high-risk. This threshold achieves an optimal balance between maintaining high detection sensitivity and minimizing unnecessary treatment burden, offering a concrete and actionable reference point for clinical decision-making.

## 4. Discussion

This study, based on a large-scale multinational longitudinal cohort, successfully developed and validated a machine learning model for predicting the progression of CKD. Compared to existing mainstream models such as KFRE, the XGBoost model constructed in this study not only showed exceptional discriminative ability (training set AUC: 0.892) and calibration but also maintained robust predictive performance in independent external cohorts from diverse ethnicities and regions (ELSA and HRS, with AUCs of 0.867 and 0.871, respectively). These findings indicate that by integrating composite indicators reflecting systemic physiological status, machine learning approaches can capture the complex nonlinear relationships underlying CKD progression, thereby facilitating the development of a risk prediction tool characterized by high accuracy and strong generalizability. The following discussion will center on the principal findings, clinical implications, limitations, and future directions of this research.

As outlined in the introduction, with the aging population and the increasing global burden of CKD, various research teams have developed numerous risk prediction models for CKD progression. A systematic review by Ramspek et al. of all such prediction models published before 2020 revealed that while these models demonstrated excellent predictive performance within specific populations for which they were developed, they generally lacked external validation and exhibited a high risk of bias [10]. Among the numerous models, only the KFRE model, the KPNW model, and the Marks model employed calibration curves to assess the consistency between the predicted probabilities and the actual observations [11,12,13], and many models only report excellent discriminative ability (e.g., a C-statistic above 0.9), which can create the illusion of “good model performance”. However, if calibration is poor, such a high-scoring model may still be unreliable in clinical practice. Among all those models, only the Marks model employed decision curve analysis to directly evaluate its clinical utility across different decision thresholds. This reflects that most studies remain at the stage of assessing “predictive accuracy” without delving into the evaluation of “clinical decision support capability”. Even if a model is accurate in prediction, its clinical application value may be significantly compromised if it fails to assist physicians in making better decisions at reasonable risk thresholds.

Currently, traditional prediction models such as the CKD-EPI equation and KFRE show significant limitations in terms of accuracy, generalizability, and early identification of rapid progressors. In contrast, advances in artificial intelligence are further enhancing the efficiency of CKD management and personalized treatment. Traditional models, which primarily rely on a limited set of variables (e.g., age, sex, eGFR, proteinuria), often experience reduced accuracy in specific patient subgroups. AI models significantly improve predictive capability by integrating multimodal data [14]. A review synthesizing nearly all artificial intelligence (AI) risk prediction models for chronic kidney disease (CKD) progression up to 2024 indicates that these models demonstrate enhanced capabilities over traditional models in handling complex data and capturing non-linear relationships, leading to superior predictive performance. However, the review also underscores their susceptibility to overfitting and emphasizes the necessity for multicenter external validation to ensure clinical applicability. Conducting a large-sample, multicenter external validation represents a primary strength of the current study [15]. Moreover, SHAP (SHapley Additive exPlanations) analysis can clearly reveal the key factors influencing predictions, thereby mitigating the distrust associated with “black-box” decision-making and assisting clinicians in better intervening in disease progression [16,17].

Chronic kidney disease (CKD) may result from a single cause, but it more commonly arises from the cumulative or concurrent effects of multiple risk factors. Its etiology encompasses various elements, including genetic, metabolic, environmental, and autoimmune factors, with significant differences observed between adults and children [1]. On the genetic front, hundreds of gene variants can cause or drive the progression of CKD, such as variations in the *PKD1* and *PKD2* genes in patients with polycystic kidney disease, and *COL4A5* gene variants in those with Alport syndrome [18]. Metabolically, diabetes and obesity are among the most common risk factors for CKD, driving disease progression by increasing the metabolic load on the kidneys. Autoimmune and inflammatory factors, such as glomerulonephritis and infections, also frequently contribute to CKD [19]. Environmental exposures linked to different occupations and latitudes, along with socioeconomic factors like poverty and inadequate healthcare resources, significantly elevate the risk of developing CKD [20,21]. Other non-modifiable factors, including aging, gender, and acute kidney injury, can also promote the progression of CKD.

Table 1 reveals significant differences in baseline characteristics across different ethnic groups. However, it is important to note that statistical significance does not necessarily imply clinical relevance. In studies with large sample sizes, even minor differences can become statistically significant, yet these variations may not have a practical impact on the progression of Chronic Kidney Disease (CKD). Therefore, by employing SHAP analysis in Figure 5, we have accurately identified the key influencing factors for CKD progression.

Through SHAP-based significance analysis, we have further validated several established conclusions. For instance, age and UACR play significant driving roles in the progression of CKD, while comorbidities such as diabetes, hypertension, and heart disease increase the risk of CKD progression. Concurrently, several intriguing new findings have emerged. First, FI incorporated in this model construction emerged as an overwhelmingly important predictor (SHAP value: 0.184), with an influence far exceeding that of traditional factors. This underscores the significant role of the frailty index, as a comprehensive systemic health indicator, in predicting diseases among the elderly. Second, other systemic indicators included in the study, such as the TyG index, AISI, and VAI, all demonstrated substantial contributions to CKD progression. These findings support the emerging perspective that CKD is not an isolated renal disease but rather a systemic disorder.

In the univariate analysis presented in Table 2, no significant difference was observed in gender distribution between the progression and non-progression groups (*p*-value = 0.841). Furthermore, in the more critical multivariate SHAP analysis (Figure 5), gender did not emerge as a significant predictor. This finding appears to contradict the prevailing view regarding gender differences in CKD progression. This discrepancy may be attributed to the possibility that the influence of gender on CKD is more pronounced in other age groups and does not act independently. Instead, its effect on CKD progression likely involves interactions with factors such as FI and comorbidities. In this study, which focused on an elderly population, comprehensive indicators like FI may have sufficiently accounted for the progression risk, allowing the machine learning model to capture and overshadow the relative influence of gender.

Among the composite indicators, FI is a powerful and independent risk factor for the progression of CKD, with a SHAP value of 0.184 and a multivariable hazard ratio of 2.35. As a core concept in geriatric medicine, it manifests as decreased physiological reserve and impaired multisystem function, leading to a diminished capacity to cope with stressors [22]. Previous studies have supported that frailty is an independent risk factor for various adverse outcomes in CKD [23,24,25]. A prospective cohort study conducted over 4 years found that frailty increased the risk of CKD progressing to end-stage kidney disease (ESKD) by 20% [26]. Another prospective cohort study based on 270,000 UK Biobank participants without baseline CKD also found that frailty status was significantly associated with the incidence of CKD, demonstrating a positive correlation with CKD risk [25]. In 2025, new study simplified frailty assessment indicators using a machine learning model and further validated that the frailty index serves as an independent predictor for renal function progression and mortality in patients with chronic kidney disease (CKD). Incorporating the frailty index into CKD prognostic models significantly enhances prediction accuracy [27,28]. The frail state can drive the progression of CKD through multiple pathophysiological mechanisms. For instance, malnutrition-induced volume depletion directly accelerates the decline of renal function [29]. Uremic toxins impair muscle and vascular function via inflammatory pathways and oxidative stress [30]. Sarcopenia, associated with frailty, worsens metabolic acidosis and protein-energy wasting (PEW), promoting renal tubular injury [26]. Furthermore, a bidirectional cycle between frailty and cognitive decline (such as “cognitive frailty”) reduces treatment adherence and exacerbates the deterioration of renal function [31].

The TyG index serves as a simple marker for assessing insulin resistance. Multiple studies have showed a positive correlation between the TyG index and the risk of CKD, with a pooled odds ratio/hazard ratio (OR/HR) of approximately 1.3–1.8. This suggests that for each 1-unit increase in the TyG index, the risk of CKD rises by 30% to 80%. This association remains significant even after adjusting for confounding factors such as obesity, blood lipids, and blood glucose levels, supporting the TyG index as an independent risk factor [32,33,34,35,36]. Insulin resistance can directly promote the progression of CKD by inducing glomerular hypertension, fibrosis, and endothelial dysfunction. It can also indirectly accelerate the decline of renal function by triggering hypertension (which increases renal vascular pressure), inflammation (elevated high-sensitivity C-reactive protein), and oxidative stress [37]. A 2024 Bayesian network analysis investigating the pathway from insulin resistance to CKD revealed a core pathway: “TyG index→Obesity→Hypertension→eGFR decline”, highlighting the important role of systemic composite indicators in predicting the progression of CKD [38].

Inflammation is a core mechanism in the progression of CKD. Persistent inflammatory response acts as a key initiating factor for renal fibrosis in CKD [39,40]. The innate and adaptive immune systems work synergistically, driving tubular injury and interstitial fibrosis through inflammatory mediators and signaling pathways, which accelerate the loss of nephrons [30,40,41,42]. Chronic inflammation and metabolic dysregulation (such as insulin resistance and lipid abnormalities) reinforce each other, exacerbating the inflammatory response and glomerular hypertension [43]. This process increases the production of reactive oxygen species (ROS) and suppresses antioxidant enzymes (e.g., superoxide dismutase), leading to mitochondrial dysfunction and DNA damage, and forming a positive feedback loop of oxidative stress [30,44]. Studies have found that CKD patients often exhibit T-cell exhaustion and impaired neutrophil chemotaxis, resulting in a persistent low-grade inflammatory state (e.g., elevated IL-6) while simultaneously increasing the risk of infections, which further accelerates kidney damage [45]. Therefore, AISI, as a composite indicator comprehensively reflecting the overall status of innate immune and inflammatory cells, can also play a significant predictive role in the progression of CKD.

Beyond systemic correlates, CKD progression is ultimately expressed as a dynamic renal trajectory, where both the slope and the variability of eGFR convey clinically meaningful information. A steeper negative eGFR slope reflects sustained structural deterioration, whereas increased visit-to-visit eGFR variability often indicates an “unstable” renal functional reserve, potentially driven by recurrent hemodynamic stress, intercurrent illness, medication effects, or short-lived subclinical insults. Importantly, higher variability is not merely measurement noise; it may represent biological vulnerability that accelerates long-term decline even when baseline eGFR is similar.

In parallel, the evolution of proteinuria provides a complementary trajectory of glomerular injury. In progressive CKD, albuminuria frequently shows an upward or stepwise-increasing trend over follow-up rather than remaining static, consistent with ongoing damage to the glomerular filtration barrier and maladaptive hyperfiltration in remaining nephrons. Clinically, a rising proteinuria trend can precede substantial eGFR loss and is actionable because it may prompt intensified renoprotective therapy and closer monitoring. This trajectory-based view is consistent with our findings that systemic frailty, insulin resistance, and inflammation (FI, TyG, and AISI) were among the most informative predictors, as these systemic states may plausibly interact with renal hemodynamics and permeability, thereby linking global vulnerability to steeper eGFR decline and worsening proteinuria.

While the present model was intentionally built on baseline predictors to maximize cross-cohort portability under heterogeneous follow-up schedules, these considerations motivate future work incorporating eGFR slope/variability features and proteinuria trend descriptors (e.g., annualized change or time-updated levels) to further enhance interpretability and clinical face validity.

The machine learning prediction model for CKD progression developed in this study aimed to mitigate common limitations observed in previous similar models. It utilized an expanded sample size to reduce overfitting and demonstrated consistent performance during internal cohort validation and external validation across diverse ethnic groups. The model’s performance and clinical utility were rigorously evaluated using decision curve analysis, calibration curves, and risk stratification methods.

However, several limitations remain: As a retrospective study, it lacks long-term follow-up data to validate the model’s dynamic predictive capability. Although the study included East Asian, European, and North American populations, its performance remains unvalidated in populations from regions with relatively limited healthcare resources, such as South America and Africa. Given the differences in genetic backgrounds and healthcare standards, the model’s predictive efficacy and applicability for these populations require further verification, which may impact algorithmic fairness and introduce group bias. The etiology, progression rate, and comorbidity profiles of chronic kidney disease may vary significantly across different age groups. The three multi-ethnic databases referenced in this study primarily comprise elderly patients, lacking data on CKD progression etiologies common in younger individuals, such as lupus nephritis. Consequently, the model may fail to capture important predictors specific to younger patients. While ensuring an adequate sample size, the common measurement indicators across the three databases were limited, omitting some early biomarkers confirmed to predict the progression to end-stage renal disease and mortality risk. This absence could affect the model’s predictive performance and limits the ability to distinguish between different types of chronic kidney disease, necessitating further subgroup analyses with more external databases in the future. To minimize survivor bias, the study endpoints were defined as progression to ESKD or all-cause mortality, which might have led to an overestimation of the CKD progression rate by including deaths from other causes.

Building upon this research, future directions could include conducting multicenter prospective studies, incorporating advanced causal inference methods (e.g., instrumental variables or doubly robust estimation) to reduce bias from unmeasured confounders, and leveraging the strengths of combined models (e.g., XGBoost + neural networks) to further enhance predictive accuracy. External validation of this model could be conducted across broader age groups and more diverse ethnic populations to further enhance its generalizability. These efforts aim to develop more generalizable and precise prediction models, ultimately improving the quality of life for global populations and reducing the worldwide burden of CKD.

## 5. Conclusions

This study successfully developed and validated a machine learning-based model for predicting CKD progression, leveraging multinational cohorts to ensure cross-ethnicity applicability. By incorporating composite indicators such as the frailty index, TyG index, and systemic inflammation markers, the model captured multidimensional pathophysiological mechanisms, outperforming traditional approaches like KFRE in discriminative ability and calibration. Key findings include the dominance of FI as a predictor, highlighting the role of systemic physiological reserve in CKD progression, and the model’s stability across diverse populations (AUC > 0.86 in external validation). The risk stratification and decision curve analyses further underscore its clinical relevance, enabling targeted interventions for high-risk groups. However, limitations such as the retrospective nature and lack of data from resource-limited regions (e.g., Africa) suggest caution in generalizability. Future research should focus on prospective multicenter studies, integration of causal inference methods, and expansion to global populations to enhance model robustness.

## Figures and Tables

**Table 1:** Baseline Characteristics of the Study Population.

Characteristics	CHARLS Training Set (*n* = 2500)	ELSA Validation Set (*n* = 1200)	HRS Validation Set (*n* = 1500)	*p*-Value
**Demographics**				
Age (years)	68.3 ± 6.2	72.1 ± 7.8	70.5 ± 7.1	<0.001
Male, *n* (%)	1285 (51.4)	572 (47.7)	712 (47.5)	0.023
BMI (kg/m^2^)	24.1 ± 3.5	26.8 ± 4.2	27.3 ± 4.6	<0.001
**Comorbidities** **, *n* (%)**				
Hypertension	1325 (53.0)	642 (53.5)	825 (55.0)	0.421
Diabetes	625 (25.0)	312 (26.0)	420 (28.0)	0.089
Heart Disease	500 (20.0)	252 (21.0)	330 (22.0)	0.267
**Laboratory Parameters**				
Hemoglobin (g/dL)	12.8 ± 1.6	13.2 ± 1.5	13.4 ± 1.7	<0.001
Albumin (g/L)	38.5 ± 4.2	39.2 ± 4.0	39.8 ± 4.3	<0.001
Blood glucose (mmol/L)	6.2 ± 1.8	5.9 ± 1.5	6.1 ± 1.7	<0.001
UACR (mg/g)	45.2 [15.8–125.6]	38.7 [12.3–98.4]	42.1 [14.2–110.3]	0.003
**Composite Indicators**				
AISI	325.6 [156.8–685.4]	298.3 [142.5–624.1]	312.8 [148.9–657.2]	0.015
TyG index	8.65 ± 0.72	8.52 ± 0.68	8.59 ± 0.71	<0.001
BRI	4.12 ± 1.25	4.85 ± 1.42	4.92 ± 1.38	<0.001
VAI	2.38 ± 1.06	2.65 ± 1.12	2.71 ± 1.15	<0.001
CMI	1.25 ± 0.58	1.32 ± 0.61	1.35 ± 0.63	<0.001
Frailty index (FI)	0.18 ± 0.08	0.21 ± 0.09	0.20 ± 0.08	<0.001
**Study Outcomes**				
CKD progression, *n* (%)	308 (12.3)	121 (10.1)	176 (11.7)	0.142

BMI: Body Mass Index; UACR: Urine Albumin-to-Creatinine Ratio; AISI: Aggregate Index of Systemic Inflammation; TyG: Triglyceride–glucose; BRI: Body Roundness Index; VAI: Visceral Adiposity Index; CMI: Cardiometabolic Index; FI: Frailty Index.

**Table 2:** Comparison of Baseline Characteristics Between Progressors and Non-progressors in the CHARLS Training Set.

Characteristic	Non-Progressors (*n* = 2192)	Progressors (*n* = 308)	*p*-Value
Demographic Characteristics			
Age (years)	67.5 ± 6.0	73.2 ± 6.8	<0.001
Male, *n* (%)	1125 (51.3)	160 (51.9)	0.841
BMI (kg/m^2^)	24.3 ± 3.4	22.8 ± 3.9	<0.001
Comorbidities, *n* (%)			
Hypertension	1125 (51.3)	200 (64.9)	<0.001
Diabetes	500 (22.8)	125 (40.6)	<0.001
Heart Disease	408 (18.6)	92 (29.9)	<0.001
Laboratory Parameters			
Hemoglobin (g/dL)	13.0 ± 1.5	11.2 ± 1.8	<0.001
Albumin (g/L)	39.1 ± 3.9	34.8 ± 4.5	<0.001
Blood glucose (mmol/L)	6.0 ± 1.6	7.5 ± 2.3	<0.001
UACR (mg/g)	32.5 [12.8–85.4]	156.8 [65.2–385.6]	<0.001
Composite Indicators			
AISI	298.4 [142.6–625.3]	512.8 [245.6–1085.2]	<0.001
TyG index	8.55 ± 0.65	9.25 ± 0.85	<0.001
BRI	4.05 ± 1.18	4.65 ± 1.52	<0.001
VAI	2.28 ± 0.98	3.05 ± 1.25	<0.001
CMI	1.18 ± 0.52	1.68 ± 0.75	<0.001
FI	0.16 ± 0.07	0.28 ± 0.09	<0.001

**Table 3:** Multivariable Cox regression.

Predictor	Unit/Contrast	Adjusted HR	95% CI	*p*-Value
Frailty index (FI)	per 0.1 increase	2.35	1.95–2.83	<0.001
Age	per 10-year increase	1.82	1.55–2.14	<0.001
UACR	per doubling (log2)	1.75	1.48–2.06	<0.001
TyG index	per 1-unit increase	1.68	1.40–2.01	<0.001
Diabetes	yes vs. no	1.42	1.18–1.71	<0.001
Hypertension	yes vs. no	1.28	1.06–1.55	0.010
Albumin	per 5 g/L increase	0.86	0.79–0.94	0.001
Hemoglobin	per 1 g/dL increase	0.92	0.88–0.97	0.002

**Table 4:** Comparative performance of tested models in internal validation (CHARLS).

Model	AUC	Accuracy	Precision	Recall	F1-Score	Brier Score
XGBoost	0.892	0.880	0.360	0.780	0.492	0.090
LightGBM	0.885	0.875	0.340	0.760	0.468	0.094
RF	0.874	0.868	0.320	0.730	0.444	0.100
SVM	0.843	0.855	0.280	0.690	0.401	0.108
LR	0.821	0.848	0.260	0.660	0.371	0.112
KDIGO	0.745	0.810	0.210	0.580	0.308	0.135

## Data Availability

The datasets used in this study are available in online repositories. The repository names and their accession numbers are available at https://charls.pku.edu.cn/en/ (CHARLS, accessed on 29 November 2025); https://datacatalogue.ukdataservice.ac.uk/studies/study/5050 (ELSA, accessed on 29 November 2025) and https://hrsdata.isr.umich.edu/data-products/public-survey-data (HRS, accessed on 29 November 2025). If you have any further questions, please reach out to the corresponding author directly.

## Supplementary Materials

The following supporting information can be downloaded at: https://www.mdpi.com/article/10.3390/jcm15020825/s1, Figure S1: External cohort’s calibration curve; Figure S2: External cohort’s decision curve; Table S1: Variable-level missingness prior to imputation (%, by cohort); Table S2: XGBoost hyperparameters and tuning grid; Table S3: Distributional comparison of variables before vs. after imputation.

## Abbreviations

The following abbreviations are used in this manuscript:

CKD	Chronic kidney disease
KFRE	the Kidney Failure Risk Equation
CHARLS	the China Health and Retirement Longitudinal Study
ELSA	the English Longitudinal Study of Aging
HRS	Health and Retirement Study
FI	Frailty index
TyG	Triglyceride–glucose
AISI	Aggregate index of systemic inflammation
AUC	Area under the curve
KDIGO	the Kidney Disease: Improving Global Outcomes
MAFLD	Metabolic dysfunction-associated fatty liver disease
CKM	Cardiovascular–kidney–metabolic
eGFR	estimated glomerular filtration rate
UACR	Urine albumin-to-creatine ratio
BRI	Body Roundness Index;
VAI	Visceral Adiposity Index;
CMI	Cardiometabolic Index;

## Appendix A

**CKD Risk Calculator (2y/5y)—with North America Recalibration**			
Mode: use internal parametric surrogate OR paste your model probability			
**Mode**	Internal		Choices: Internal/Paste_p (dropdown)
**Age (years)**	70		Numeric
**Sex (1 = Male, 0 = Female)**	1		0 or 1
**eGFR (mL/min/1.73 m^2^)**	45		Numeric
**UACR (mg/g)**	100		Numeric mg/g
**Diabetes (1/0)**	0		Optional
**Hypertension (1/0)**	1		Optional
**North America intercept shift (logit)—2y**	0		Set per site calibration
**North America intercept shift (logit)—5y**	0		Set per site calibration
**(Optional) Paste base p_model_2y (0–1)**			Used only when Mode = Paste_p
**(Optional) Paste base p_model_5y (0–1)**			Used only when Mode = Paste_p
**Transforms**			
x_age = (Age-65)/10	0.5		
x_egfr = (60—eGFR)/10	1.5		
x_lnacr = LN(UACR + 1)	4.615120517		
**Base probabilities (before NA intercept shift)**		**Recalibrated probabilities (North America shift)**	
base_p_2y	19.166%	p_NA_2y	19.166%
base_p_5y	40.686%	p_NA_5y	40.686%
**Subgroup hints**			
UACR category	A2 (30–300)		
G-stage by eGFR	G3a (45–59)		
	Notes: (1) If Mode = Paste_p, outputs depend ONLY on cells B12/B13 (base p_model). Inputs above won’t change the risk. (2) To make inputs drive results, set Mode = Internal. (3) North America recalibration uses logit shift (B12 for 2y, B13 for 5y). Default 0.00. (4) Ensure Excel calculation is set to Automatic (Formulas -> Calculation Options -> Automatic). (5) Coefficients can be edited in the ‘Coefficients’ sheet.		

## Appendix B

**Scenario**	**Cohort**	**Horizon**	**N**	**Events**	**Event_Rate**	**AUC**	**Brier**	**Calib_Intercept**	**Calib_Slope**
main	CHARLS	2y	2500	132	0.053	0.868	0.049	0.191	0.034
main	CHARLS	5y	2500	312	0.125	0.891	0.075	0.346	0.069
main	ELSA	2y	1200	50	0.042	0.853	0.037	0.174	0.031
main	ELSA	5y	1200	104	0.087	0.867	0.062	0.273	0.053
main	HRS	2y	1500	83	0.055	0.835	0.054	0.189	0.032
main	HRS	5y	1500	189	0.126	0.867	0.082	0.354	0.07
S2_exclude_any_AKI	CHARLS	2y	2315	120	0.052	0.867	0.048	0.188	0.034
S2_exclude_any_AKI	CHARLS	5y	2315	290	0.125	0.891	0.075	0.349	0.07
S2_exclude_any_AKI	ELSA	2y	1084	45	0.042	0.851	0.037	0.173	0.03
S2_exclude_any_AKI	ELSA	5y	1084	95	0.088	0.878	0.061	0.282	0.055
S2_exclude_any_AKI	HRS	2y	1364	82	0.06	0.837	0.056	0.206	0.035
S2_exclude_any_AKI	HRS	5y	1364	177	0.13	0.868	0.083	0.364	0.072
