# Supplementary material for: A Cross-Ethnicity Validated Machine Learning Model for the Progression of Chronic Kidney Disease in Individuals over 50 Years Old
Source: J Clin Med. 2026 Jan 20;15(2):825. doi: 10.3390/jcm15020825 (PMC12841880; doi:10.3390/jcm15020825)
Supplement: Supplementary file 1 [file jcm-15-00825-s001.zip › jcm-4044394-supplementary.pdf]

Figure S1a

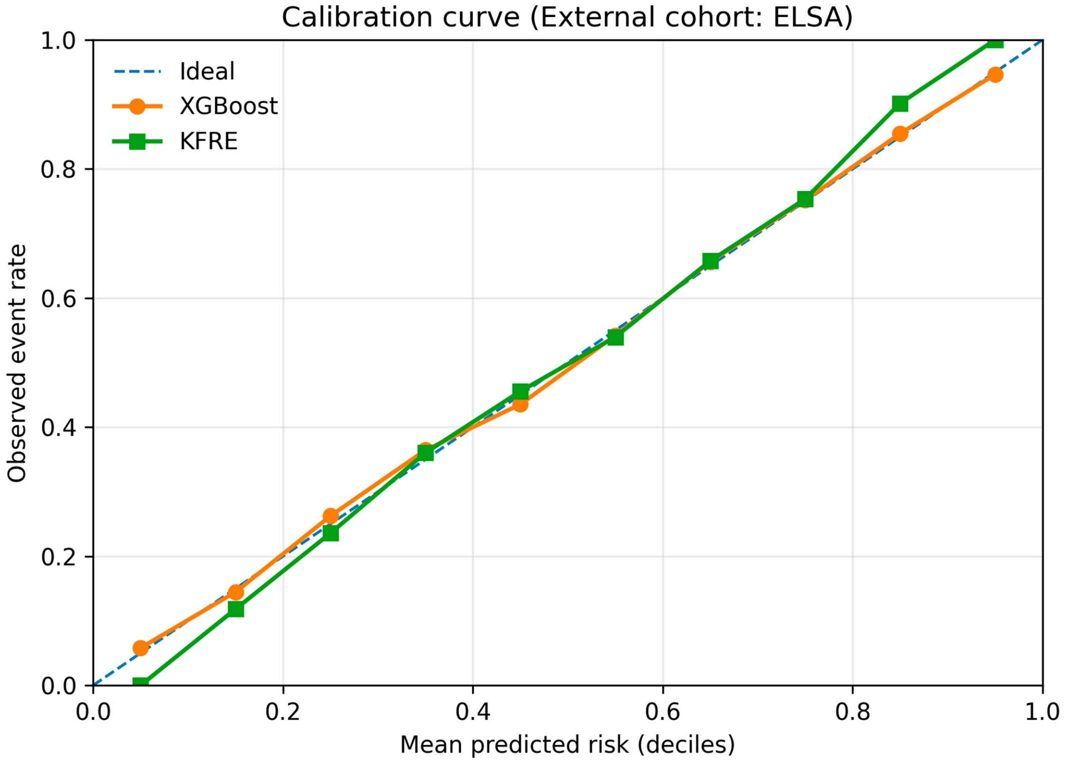


Figure S1b

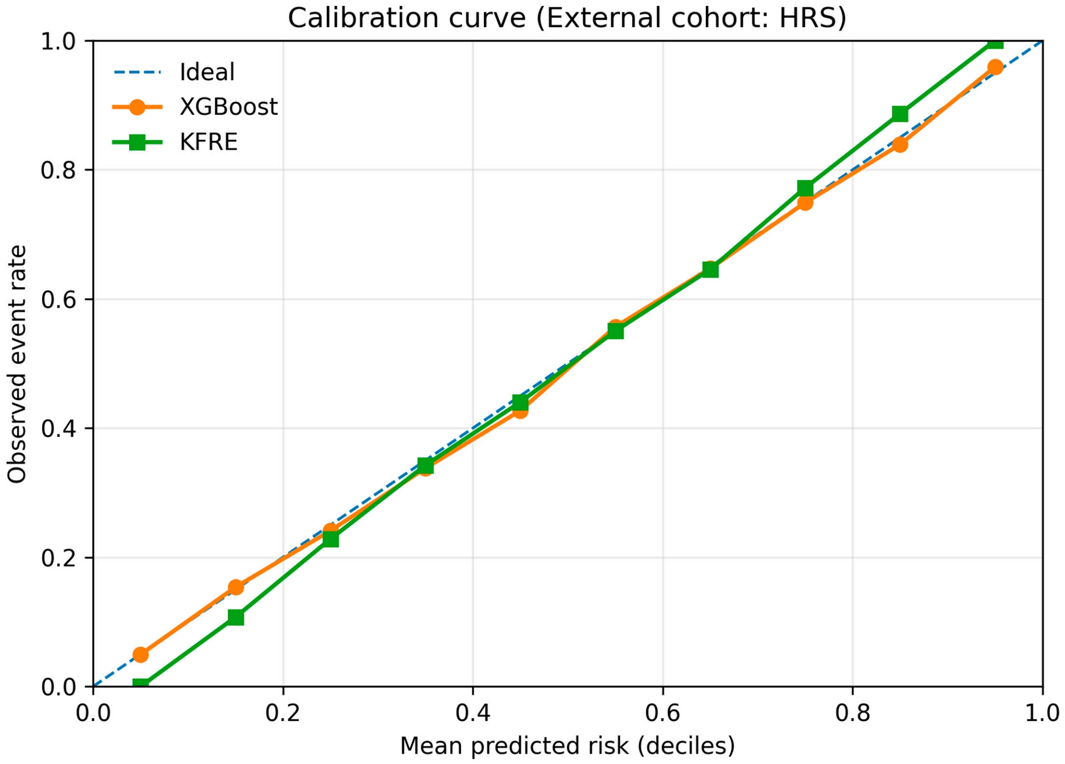

Figure S2a

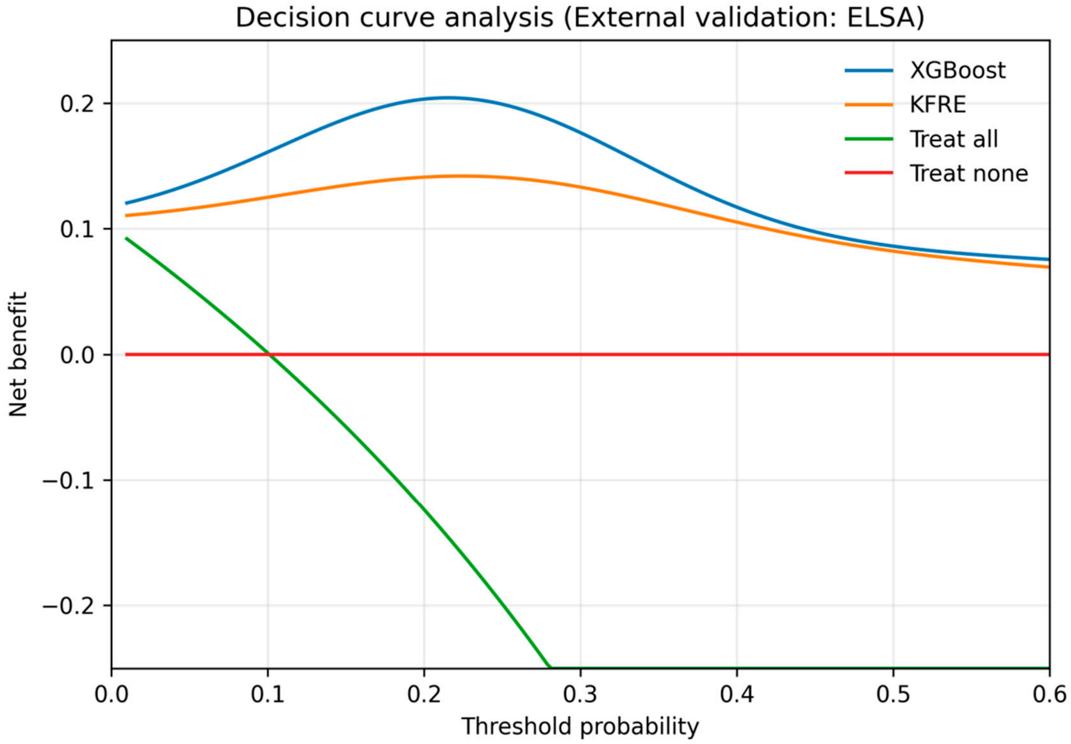


Figure S2b

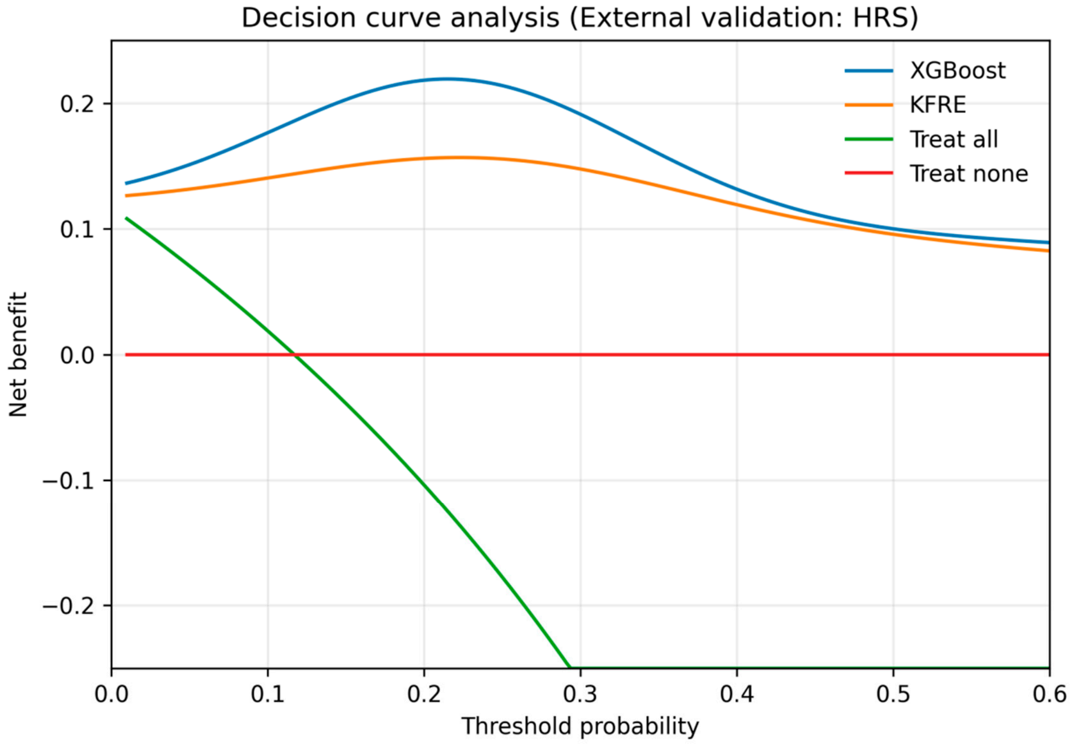

Table S1. Variable-level missingness prior to imputation (%, by cohort)

| Variable | CHARLS (n=2,500) | ELSA (n=1,200) | HRS (n=1,500) | Overall (N=5,200) |
|---|---|---|---|---|
| Age (years) | 0 (0.00%) | 0 (0.00%) | 0 (0.00%) | 0 (0.00%) |
| Male (sex) | 0 (0.00%) | 0 (0.00%) | 0 (0.00%) | 0 (0.00%) |
| BMI (kg/m²) | 10 (0.40%) | 6 (0.50%) | 9 (0.60%) | 25 (0.48%) |
| Hypertension (yes/no) | 5 (0.20%) | 2 (0.17%) | 4 (0.27%) | 11 (0.21%) |
| Diabetes (yes/no) | 5 (0.20%) | 2 (0.17%) | 4 (0.27%) | 11 (0.21%) |
| Heart disease (yes/no) | 8 (0.32%) | 4 (0.33%) | 6 (0.40%) | 18 (0.35%) |
| Hemoglobin (g/dL) | 70 (2.80%) | 36 (3.00%) | 39 (2.60%) | 145 (2.79%) |
| Albumin (g/L) | 95 (3.80%) | 50 (4.17%) | 54 (3.60%) | 199 (3.83%) |
| Fasting blood glucose (mmol/L) | 45 (1.80%) | 19 (1.58%) | 26 (1.73%) | 90 (1.73%) |
| UACR (mg/g) | 112 (4.48%) | 46 (3.83%) | 63 (4.20%) | 221 (4.25%) |
| AISI | 75 (3.00%) | 31 (2.58%) | 42 (2.80%) | 148 (2.85%) |
| TyG index | 52 (2.08%) | 22 (1.83%) | 30 (2.00%) | 104 (2.00%) |
| BRI | 25 (1.00%) | 10 (0.83%) | 14 (0.93%) | 49 (0.94%) |
| VAI | 65 (2.60%) | 26 (2.17%) | 36 (2.40%) | 127 (2.44%) |
| CMI | 72 (2.88%) | 30 (2.50%) | 41 (2.73%) | 143 (2.75%) |
| Frailty index (FI) | 30 (1.20%) | 18 (1.50%) | 20 (1.33%) | 68 (1.31%) |

Table S2. XGBoost hyperparameters and tuning grid

| Parameter | Search grid (grid search+5-fold CV) | Final value | Notes |
|---|---|---|---|
| booster | gbtree | gbtree | Tree-based boosting |
| objective | binary:logistic | binary:logistic | Binary classification (CKD progression) |
| eval_metric | auc | auc | Optimized for discrimination |
| n_estimators | [200, 400, 600, 800] | 600 | Number of boosting rounds |
| learning_rate (eta) | [0.01, 0.03, 0.05, 0.1] | 0.05 | Shrinkage to reduce overfitting |
| max_depth | [3, 4, 5, 6] | 4 | Tree depth |
| min_child_weight | [1, 3, 5, 7] | 5 | Minimum sum of instance weight needed in a child |
| subsample | [0.6, 0.8, 1.0] | 0.8 | Row sampling |
| colsample_bytree | [0.6, 0.8, 1.0] | 0.8 | Column sampling per tree |
| gamma | [0, 0.5, 1.0] | 0 | Minimum loss reduction to make a split |
| reg_alpha (L1) | [0, 0.1, 0.5] | 0.1 | L1 regularization |
| reg_lambda (L2) | [0.5, 1.0, 2.0] | 1.0 | L2 regularization |
| scale_pos_weight | [1, 3, 5, 7] | 7 | Class-imbalance handling (approx. neg/pos in training set) |
| random_state | fixed | 42 | Reproducibility |
| n_jobs | fixed | -1 | Parallel computation |

Table S3. Distributional comparison of variables before vs. after imputation

| Variable | Pre-imputation (complete cases) | Post-imputation (full analytic set) | Δ(post−pre) | SMD |
|---|---|---|---|---|
| BMI (kg/m²) | 25.62 ± 4.08 | 25.65 ± 4.07 | +0.03 | +0.01 |
| Hemoglobin (g/dL) | 13.05 ± 1.64 | 13.07 ± 1.63 | +0.02 | +0.01 |
| Albumin (g/L) | 39.02 ± 4.21 | 39.04 ± 4.20 | +0.02 | +0.00 |
| Fasting blood glucose (mmol/L) | 6.11 ± 1.69 | 6.10 ± 1.68 | -0.01 | -0.01 |
| UACR (mg/g) | 44.0 [15.2–120.0] | 43.5 [14.8–118.5] | -0.5 (median) | -0.01 |
| AISI | 322 [152–670] | 318 [150–660] | -4 (median) | -0.01 |
| TyG index | 8.60 ± 0.71 | 8.60 ± 0.71 | +0.00 | +0.00 |
| BRI | 4.52 ± 1.34 | 4.52 ± 1.33 | +0.00 | +0.00 |
| VAI | 2.54 ± 1.12 | 2.54 ± 1.11 | +0.00 | +0.00 |
| CMI | 1.30 ± 0.62 | 1.30 ± 0.61 | +0.00 | +0.00 |
| Frailty index (FI) | 0.193 ± 0.085 | 0.193 ± 0.084 | +0.000 | +0.00 |
